# Rice Genotype Differences in Tolerance of Zinc-Deficient Soils: Evidence for the Importance of Root-Induced Changes in the Rhizosphere

**DOI:** 10.3389/fpls.2015.01160

**Published:** 2016-01-11

**Authors:** Asako Mori, Guy J. D. Kirk, Jae-Sung Lee, Mark J. Morete, Amrit K. Nanda, Sarah E. Johnson-Beebout, Matthias Wissuwa

**Affiliations:** ^1^Crop Production and Environment Division, Japan International Research Center for Agricultural SciencesTsukuba, Japan; ^2^Cranfield Soil and Agrifood Institute, School of Energy, Environment and Agrifood, Cranfield UniversityCranfield, UK; ^3^Crop and Environmental Sciences Division, International Rice Research InstituteMetro Manila, Philippines

**Keywords:** genotype, crown roots, root surface area, root efficiency, rhizosphere, micronutrient

## Abstract

Zinc (Zn) deficiency is a major constraint to rice production and Zn is also often deficient in humans with rice-based diets. Efforts to breed more Zn-efficient rice are constrained by poor understanding of the mechanisms of tolerance to deficiency. Here we assess the contributions of root growth and root Zn uptake efficiency, and we seek to explain the results in terms of specific mechanisms. We made a field experiment in a highly Zn-deficient rice soil in the Philippines with deficiency-tolerant and -sensitive genotypes, and measured growth, Zn uptake and root development. We also measured the effect of planting density. Tolerant genotypes produced more crown roots per plant and had greater uptake rates per unit root surface area; the latter was at least as important as root number to overall tolerance. Tolerant and sensitive genotypes took up more Zn per plant at greater planting densities. The greater uptake per unit root surface area, and the planting density effect can only be explained by root-induced changes in the rhizosphere, either solubilizing Zn, or neutralizing a toxin that impedes Zn uptake (possibly ${\text{HCO}}_{3}^{-}$ or Fe^2+^), or both. Traits for these and crown root number are potential breeding targets.

## Introduction

Decreased crop growth and yield under low soil zinc (Zn) availability is a widespread problem in rice because of the particular chemistry of submerged paddy soils (Dobermann and Fairhurst, 2000). Reducing conditions in submerged soils tend to make Zn insoluble and therefore unavailable to plants, even though total amounts are generally many orders of magnitude greater than crop requirements. Since Zn binds more than 500 plant proteins (Graham et al., 2012), deficiency causes many symptoms, including chlorosis, bronzing, resetting, and goblet leaves (Broadley et al., 2007). Deficiency in the crop also causes deficiency in human populations with rice-based diets, and human Zn deficiency is one of the most widespread and serious health problems in developing countries (WHO, 2002; Gibson, 2006; Hess et al., 2009; Graham et al., 2012). Dietary and crop Zn deficiency are inevitably linked in areas with Zn-deficient soils, as in large parts of south and south-east Asia where rice is the staple (Maclean et al., 2013).

There is large variation in the rice germplasm in tolerance of Zn-deficient soils (Quijano-Guerta et al., 2002; Wissuwa et al., 2006) and in the ability to concentrate Zn in grains (Gregorio, 2002; Wissuwa et al., 2008; Impa et al., 2013). But understanding of the mechanisms underlying genotype differences is poor, and this constrains efforts to breed more Zn-efficinet rice with high grain Zn contents. Genotypes with large grain Zn contents in soils without Zn constraints tend not to produce large grain Zn contents under deficiency, and under at least moderate deficiency, high grain Zn may be at the cost of reduced grain yield (Wissuwa et al., 2008). Enhancing the Zn uptake capacity of rice genotypes will therefore be crucial to increasing grain contents.

The mechanisms behind low Zn tolerance in rice have been shown to be related to root traits (Rose et al., 2013). Possible mechanisms include:

1. maintenance of new root growth through more efficient internal use of Zn (Widodo et al., 2010; Rose et al., 2011);
2. maintenance of root growth through tolerance of high bicarbonate (${\text{HCO}}_{3}^{-}$) stress, which is often linked to Zn deficiency, especially in calcareous soils (Yang et al., 1994; Hajiboland et al., 2005; Rose et al., 2011, 2012);
3. root-induced changes in the soil neutralizing toxins such as ${\text{HCO}}_{3}^{-}$ or making Zn more soluble by the release of acidifying agents (Kirk and Bajita, 1995) or Zn-chelating phytosiderophores (Arnold et al., 2010; Widodo et al., 2010; Ptashnyk et al., 2011), or soil CO_2_ uptake (Begg et al., 1994).

**Figure 1:**
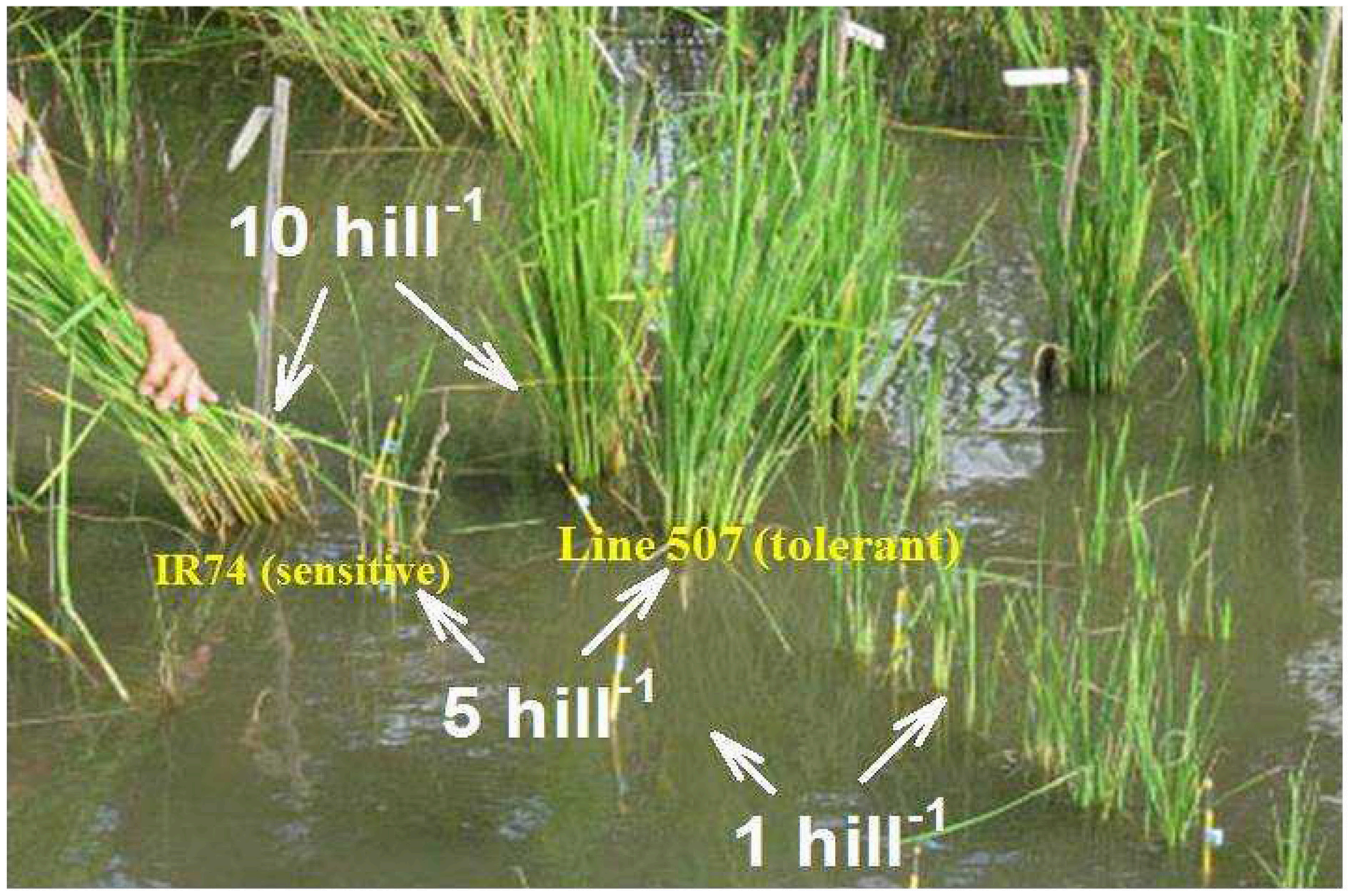
**Rice growth in a strongly Zn-deficient soil is improved by closer planting**. The right-most row is a Zn-deficiency tolerant variety (line 507); the left-most row is the sensitive variety IR74. There are three planting densities as indicated.

Indirect evidence for the importance of root-induced changes in the rhizosphere was provided by Hoffland et al. (2006) who found that, in a strongly Zn-deficient rice soil, Zn uptake per plant increased with increasing planting density to the extent that growth of rice genotypes that strongly differed in growth at low planting density was similar at high density (Figure 1). Hoffland et al. explained this effect by concentration-dependent solubilization of Zn in the rhizosphere, and Ptashnyk et al. (2011) showed with a mathematical model that planting density effects could be accounted for with realistic rates of root-induced solubilization. However, this evidence remains circumstantial.

Although the mechanisms are not yet fully understood, it has been shown repeatedly that superior field performance of genotypes is associated with better root development (Widodo et al., 2010; Rose et al., 2013). It is possible that small increases in Zn uptake as a result of root-induced changes in the rhizosphere drive better root development under Zn deficiency. However, it is equally feasible that differences in root development under Zn deficiency are not related to plant Zn content but due to differences in stress response or internal efficiency, and that better root development subsequently leads to better Zn uptake. Such questions of cause and effect are difficult to resolve because small changes in one component of the system—for example, ability to produce more root length for a given quantity of Zn taken up—can have large net effects through positive feedback loops (Wissuwa, 2003). The situation is further complicated for Zn in rice because multiple stress tolerance mechanisms appear to be involved, as shown by studies of Wissuwa et al. (2006) with Zn-efficient recombinant inbred lines (RILs) which identified four quantitative trait loci (QTLs) associated with high plant mortality and four QTLs associated with leaf bronzing, one of which was linked to both symptoms.

However, from a plant breeding perspective, it is important to resolve whether traits are direct causes of tolerance or merely indirect effects. Causative factors can be utilized in genotype selection, either via phenotyping for the causative trait, or through identification of linked loci and markers and subsequent marker assisted selection (MAS). The objectives of this study were to establish whether superior Zn uptake in Zn-deficient soil by tolerant rice genotypes is causally related to genotypic differences in crown root development or Zn uptake efficiency. To this end, we conducted a field study with several sensitive and tolerant genotypes grown under Zn-deficient conditions, and measured root development, and Zn uptake over time. We also investigated further the planting-density effect described above.

## Materials and methods

### Experiment 1: Genotype differences in growth and Zn uptake

A field experiment was carried out at the International Rice Research Institute, Los Baños, Laguna, Philippines in the dry season of 2014 (February–March). Experimental plots were two concrete tanks (8 × 16 × 0.3 m deep) containing severely Zn-deficient soil from Tiaong, Quezon province, Philippines. The soil is a Hydraquent (USDA Soil Taxonomy) with 42% clay, 40% silt, pH (aerobic in H_2_O) 8.5, CEC 9.0 cmol_c_ kg^−1^, organic C content 73 g kg^−1^, and carbonate content 96 g kg^−1^. The available Zn content (0.1 M HCl extraction) of the submerged soil at transplanting of the crop was 0.1 μg g^−1^ (Izquierdo et al., 2015). During the plot preparations, a +Zn plot adjacent to the –Zn plot received 20 kg Zn ha^−1^, and both plots received the standard recommended dose of NPK (14-14-14) at a rate of 136 kg ha^−1^.

Five genotypes were used: two tolerant of low Zn conditions (A69-1 and IR55179-3B-11–3, hereafter IR55179) and three sensitive to it (IR26, IR64, and TCC266) according to Impa et al. (2013) and our preliminary results. Seeds of the five genotypes were germinated by soaking in water, and the seedlings were then grown in a seedling tray for 20 d. The soil was flooded and puddled, and, after 21 d, the 20-d old seedlings were transplanted at a spacing of 20 cm within and between rows, with one plant per hill. Four replicates were made of each genotype and Zn treatment in a randomized block design. Individual plants were sampled 7, 14, 21, and 28 DAT. At each sampling, six individual plants were pulled out from the soil, leaf number and plant height were measured, and shoots and roots were separated, washing the roots with tap water to remove adhering soil, and the numbers of crown roots counted.

A subsample of two plants was then processed for root scanning. The experimental soil is sufficiently loose when water-saturated that a complete separation of roots from soil is possible with minimal damage to fine roots. Washed roots were stained in “Dylon Ebony Black” (Dylon International) to obtain better contrast from the background in scanned images. The roots were arranged on a transparent plastic tray with as little overlap as possible, and scanned using a flatbed top-lit scanner (EPSON STD 4800). Each scanned image was analyzed using WinRHIZO (Version 2008a, Regent Instruments Inc.). Three root diameter classes were distinguished, roughly corresponding to fine lateral roots (< 0.15 mm), lateral roots (0.15–0.40 mm), and main crown roots (>0.4 mm). The shoot and root samples including scanned roots were oven-dried at 60°C and weighed.

### Experiment 2: Effect of planting density

In addition to the main experiment, in which a single seedling was transplanted per hill, a second experiment was conducted with 1, 2, 4, or 8 seedlings transplanted close together in each hill with four replications. The experiment was made in the same plots as the main experiment following identical procedures except that transplanting was done 1 week later. The genotypes were A69-1, IR55179, IR26, IR64, and TCC266, as in the main experiment. The plants were sampled at 21 and 28 DAT by removing whole hills from the soil. Roots were washed with tap water and multiple seedlings per hill were separated into individual plants for measurements of plant height, leaf number, root length, and crown root number. Shoots and roots were oven-dried at 60°C and weighed separately.

### Experiment 3: Seedling Zn efficiency and redistribution

To explore the reasons for better crown root development in the tolerant genotypes, and redistribution of shoot Zn to roots, we conducted a nutrient solution experiment. Seedlings of tolerant and sensitive genotypes were raised in half-strength Yoshida solution (Yoshida et al., 1976) without Zn in 12-L acid-washed plastic containers. The experiment was conducted in a growth chamber at 30/25°C day/night temperature at 50% relative humidity and 12 h light period. At 12 days after sowing (DAS) one set of seedlings was sampled and from a second set roots were removed by cutting with scissors at the crown. These seedlings were replanted in fresh half-strength Yoshida solution containing no Zn but 1 mM KHCO_3_, raising the solution pH to 7.4. After a further 13 d, the plants were harvested and shoot and root dry weights and Zn contents determined.

### Tissue Zn analysis

Shoot samples were ground and 0.5 g portions were soaked in 1 N HCl for 24 h. The extract was filtered and the filtrate Zn concentration determined with a flame atomic absorption spectrometer (PerkinElmer AAnalyst 200). Zinc concentrations were only determined on shoot tissue; attempts to do so in roots were not successful due to the adherence of soil material to root surfaces. Despite repeated careful washing, root Zn concentrations were unrealistically high (30–100 μg Zn g^−1^) and far more variable than shoot Zn, indicating contamination with soil, as confirmed by the presence of high aluminum concentrations in extracts. This is a particular problem in puddled, flooded, clayey soils of the sort in our experiments. Attempts to quantify Zn in roots were therefore abandoned and root Zn concentrations were estimated based on typical root to shoot Zn ratios observed in nutrient solution experiments. In these experiments root Zn concentrations were typically 70% higher than shoot Zn concentrations (Figure S1). Throughout the manuscript total Zn uptake is taken as represented by measured shoot Zn content plus estimated root Zn content. For tissue Zn analysis of samples from the nutrient solution experiment, total root biomass (around 50 mg) and 100 mg of ground shoot biomass were digested in 0.5 mL HNO_3_ and 2 mL H_2_O_2_ using a microwave digestion system (MLS 1200 Mega, Milestone) following procedures of Miller (1998). Digests were diluted to 10 mL and Zn concentrations determined using inductively coupled plasma atomic emission spectrophotometry (ICPE-9000, Shimadzu, Kyoto, Japan).

### Data analysis

Genotype effects were analyzed with the GLM procedure using the software Statistix (Version 9). ANOVA-protected Least Significant Differences (LSD) were used to test for significant differences between genotypes or groups of genotypes at each sampling time or planting density.

To estimate the contribution of genotypic differences in root efficiency to Zn uptake, we used the data on cumulative total Zn uptake and root surface area over time to calculate uptake rates per unit root surface area as follows. We fitted the following exponential equations to the data using the “solver” function in Microsoft Excel and minimizing the sum of squares between measured and estimated values:

(1) $$\begin{array}{l} U=a\text{ exp}\left(bt\right)-c \end{array}$$

and

(2) $$\begin{array}{l} RSA=p\text{ exp}\left(qt\right)-s \end{array}$$

where *U* is the Zn uptake per plant at time *t, RSA* is the root surface area per plant at *t*, and the other symbols are coefficients. Differentiating Equation (1) gives for the rate of change in uptake at *t*:

(3) $$\begin{array}{l} dU∕dt\;=\;ab\text{ exp}\left(bt\right) \end{array}$$

We then calculate the rate of uptake per unit root surface area—which we define as the root efficiency, *RE*—at any particular time from

(4) $$\begin{array}{l} RE=\left(dU∕dt\right)∕RSA \end{array}$$

The value of *RE* will vary with the plant's internal Zn status as well as with the Zn concentration in the soil solution at the root surface, which may be altered by root-induced changes in the soil. So this term lumps together the effects of all mechanisms that increase the rate of Zn uptake into the plant per unit *RSA*.

The fits to Equations (1) and (2) were made by minimizing the sum of squares of differences using the Solver routine in Microsoft Excel with the GRG Linear engine.

## Results

### Genotype differences in growth and Zn uptake

The comparison in Table 1 of shoot biomass in the two Zn treatments shows that Zn was the main growth-limiting factor. Between 7 and 28 days after transplanting (DAT) average shoot biomass increased 10-fold in the –Zn plots compared to 50-fold in the +Zn plots. The difference was highly significant from 14 DAT. That Zn was the growth-limiting factor was confirmed by the sharp decline of shoot Zn concentrations below the critical threshold level of 15 μg Zn g^−1^ (Dobermann and Fairhurst, 2000) from 14 DAT onwards, whereas Zn concentrations remained above 20 μg Zn g^−1^ in the +Zn treatment (Table 1). Since the objective of this study is to assess the relative contributions of root growth and root efficiency to Zn uptake under Zn-deficient conditions, the remaining part of the study focuses on the –Zn treatment.

**Table 1:** **Zinc treatment effects on total plant dry weight (DW) and shoot Zn concentrations, measured at weekly intervals between 7 and 28 days after transplanting (DAT), averaged over the genotypes**.

**Treatment**	**7 DAT**	**15 DAT**	**21 DAT**	**28 DAT**
**TOTAL PLANT DW (mg plant^−1^)**				
–Zn	60.3	144.7	262.4	608.7
	(2.3)	(6.9)	(22.7)	(81.0)
+Zn	58.7	218.8	709.2	2549.9
	(2.2)	(8.3)	(47.7)	(210.0)
**SHOOT Zn CONCENTRATION (μg g^−1^)**				
–Zn	31.1	11.3	11.9	12.8
	(1.7)	(0.3)	(0.5)	(0.5)
+Zn	37.9	23.6	24.7	22.9
	(1.3)	(0.5)	(1.0)	(0.4)

*Values in brackets are standard errors of means*.

**Figure 2:**
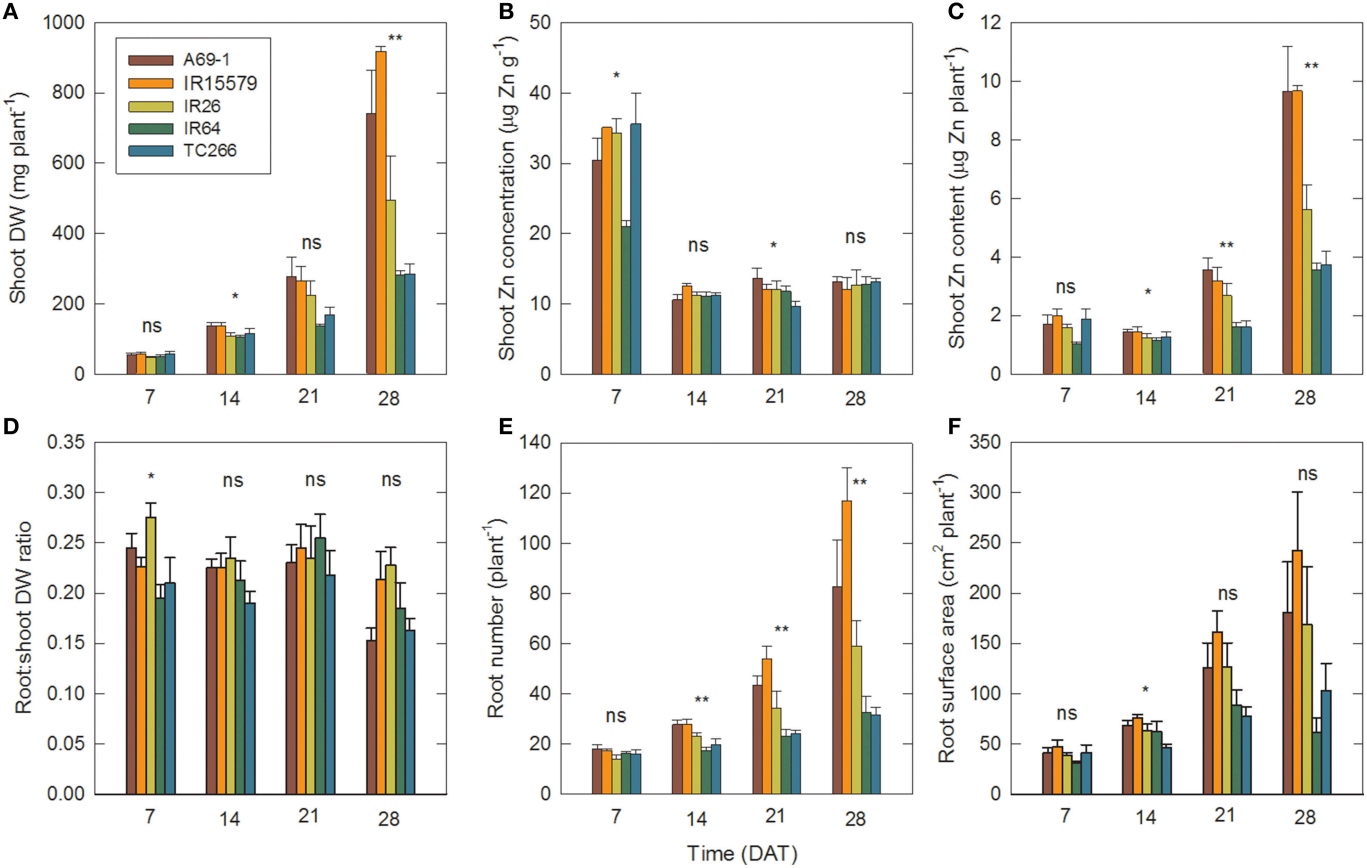
**Genotypes that differ in tolerance to Zn deficiency also differ in root number and root surface area**. Time courses of **(A)** shoot dry weight (DW), **(B)** shoot Zn concentration, **(C)** shoot Zn content (i.e., DW × concentration), **(D)** root:shoot DW ratio, **(E)** root number, and **(F)** root surface area in individual genotypes in the –Zn plots. Data are means ± SE (*n* = 4). ns, ^*, **^ = genotype differences not signif., signif. at *P* ≤ 0.05, signif. at *P* ≤ 0.01. DAT = days after transplanting.

In this Zn-deficient treatment genotypes A69-1 and IR55179 had the greatest total dry weight, shoot Zn content, and root number, whereas genotypes IR64 and TCC266 were consistently low in these (Figure 2). Shoot Zn contents at 28 DAT in A69-1 and IR55179 were roughly twice those in the sensitive genotypes. These results are consistent with earlier observations on this set of genotypes (Impa and Wissuwa, unpublished) and the genotypes can therefore be divided into Zn-deficiency tolerant (A69-1 and IR55179) and sensitive (IR26, IR64, TCC266) groups. Interestingly these groups did not separate for shoot Zn concentrations; all genotypes dropped below the critical level of 15 μg Zn g^−1^ between 7 and 14 DAT and remained at that low level throughout.

**Figure 3:**
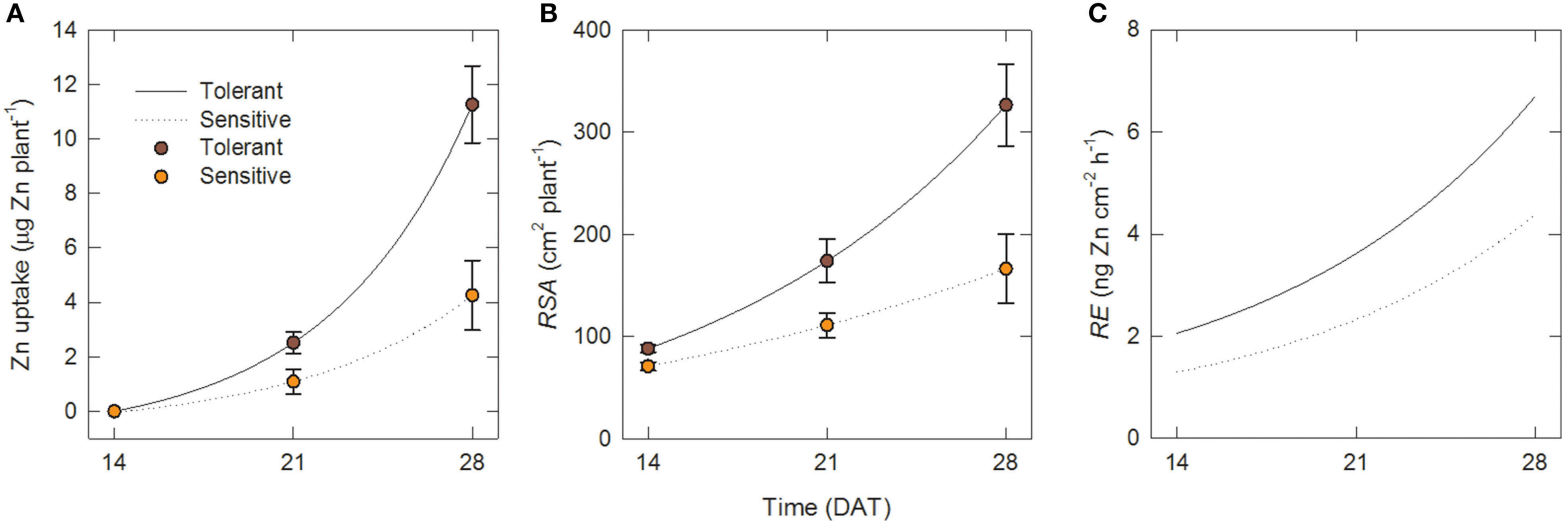
**Zinc uptake per unit root surface area increases over time and is greater in tolerant genotypes**. Time courses over 14–28 DAT in tolerant and sensitive genotype groups of **(A)** total Zn uptake per plant, **(B)** root surface area per plant and **(C)** uptake rate per unti root surface area (root efficiency). Points in **(A)** and **(B)** are measured data [means ± SE (*n* = 4)]; lines are fits to Equations (1) and (2). Lines in **(C)** are calculated from Equations (2) to (4).

Between 7 and 14 DAT plant biomass more than doubled (Figure 2A, Table 1), but since that was accompanied by a sharp drop in shoot Zn concentrations, there was a minor drop in Zn content (Figure 2C), indicating that plant growth at this point was entirely supported by Zn retranslocation and not by uptake. While the slight decrease in shoot Zn content occurred in both tolerant and sensitive groups, there were already significantly larger root surface area and root number in the tolerant than the sensitive group at 14 DAT (88.0 and 70.7 cm^2^ plant^−1^, and 26.5 and 20.1 crown roots plant^−1^, respectively). Group differences in root surface area further increased between 14–21 and 21–28 DAT, and this was accompanied by an uptake of Zn into the shoot of roughly similar proportions. Thus, with 22% greater *RSA* at 14 DAT and 45% greater *RSA* at 21 DAT, tolerant genotypes achieved 125% greater Zn uptake by 21 DAT (Figure 3). The difference in *RSA* increased further from 45 to 95% by 28 DAT and this was accompanied by 174% greater Zn uptake in the tolerant group.

Pearson's correlation coefficients indicated a close association between biomass traits and Zn content in the –Zn plots (Table 2). This was to be expected given that genotypic variation for shoot Zn concentration was too small to have an effect. There was no correlation between shoot Zn content and concentration. Of traits without an expected auto-correlation with Zn content, root biomass, root number, and *RSA* were most closely associated with Zn content, whereas the proportion of fine roots (< 150 μm diameter) and the root to shoot DW ratio were not correlated with Zn content. There were also greater crown root numbers in the tolerant group in the +Zn plots (Figure S2).

**Table 2:** **Pearson's correlation coefficients for data from the –Zn plots**.

	**Total DW**	**Shoot DW**	**Root DW**	**Shoot Zn cont**.	**Shoot Zn conc**.	**Root number**	***RSA***	**% fine roots**	**Root: shoot ratio**
Total DW	–	**0.99**	**0.97**	**0.89**	–0.01	**0.72**	**0.81**	–0.38	–0.17
Shoot DW	**0.99**	–	**0.94**	**0.87**	–0.06	**0.70**	**0.80**	–0.37	–0.24
Root DW	**0.95**	**0.90**	–	**0.91**	0.14	**0.75**	**0.78**	–0.41	0.08
Shoot Zn cont.	**0.91**	**0.94**	**0.77**	–	0.43	**0.72**	**0.69**	–0.29	0.01
Shoot Zn conc.	**–0.47**	–0.42	**–0.55**	–0.14	–	0.20	–0.07	0.1	**0.50**
Root number	**0.90**	**0.89**	**0.85**	**0.82**	–0.40	–	**0.75**	–0.34	0.05
*RSA*	**0.92**	**0.89**	**0.93**	**0.72**	**–0.58**	**0.84**	–	–0.45	–0.16
% fine roots	**–0.63**	**–0.63**	**–0.59**	**–0.59**	0.27	–0.37	**–0.58**	–	–0.06
Root:shoot ratio	0.27	0.16	**0.52**	0.01	–0.42	0.22	0.38	–0.24	–

*Values above the diagonal are for 21 DAT, below for 28 DAT. Significant correlations are indicated in bold letters (P < 0.05 at r > 0.46)*.

### Root efficiency

The results in Figure 2 suggest growth in the –Zn plots followed an exponential pattern. In this section we fit exponential functions (Equations1 and 2) to the data for root growth and Zn uptake, and then calculate values of the root efficiency (*RE*, Equation 4) for the Zn-deficiency tolerant (A69-1 and IR55179) and sensitive (IR26, IR64, TCC266) genotype groupings. Since we detected no Zn uptake from 0 to 14 DAT, we only use the data for 14–28 DAT.

The fits to Equations (1) and (2) for total Zn uptake, *U* and *RSA* were (a) for the tolerant group:

(5) $$\begin{array}{rcl} U & = & 1.09\text{ exp}\left(0.171t\right)-1.09\;RSS=4.5\times 10^{-8} \end{array}$$

(6) $$\begin{array}{rcl} RSA & = & 110.2\text{ exp}\left(0.082t\right)-22.2\;RSS=4.3\times 10^{-7} \end{array}$$

and (b) for the sensitive group:

(7) $$\begin{array}{rcl} U & = & 0.64\text{ exp}\left(0.150t\right)-0.63\;RSS=6.7\times 10^{-12} \end{array}$$

(8) $$\begin{array}{rcl} RSA & = & 108.1\text{ exp}\left(0.045t\right)-37.4\;RSS=1.7\times 10^{-7} \end{array}$$

where *U* is in μg Zn plant^−1^, *RSA* in cm^2^ plant^−1^, and *t* in days, and *RSS* is the residual sum of squares. The lines for these fits are shown in Figures 3A,B together with the experimental data.

The corresponding values of the root efficiency, *RE*, calculated with Equation (4), are shown in Figure 3C. Figure 3C shows that at all times, *RE* values were larger in the tolerant group and in both genotype groups they increased over time. Hence the superior Zn uptake of tolerant genotypes was not only due to better root growth but also to about 55% greater Zn uptake per unit root surface area.

**Figure 4:**
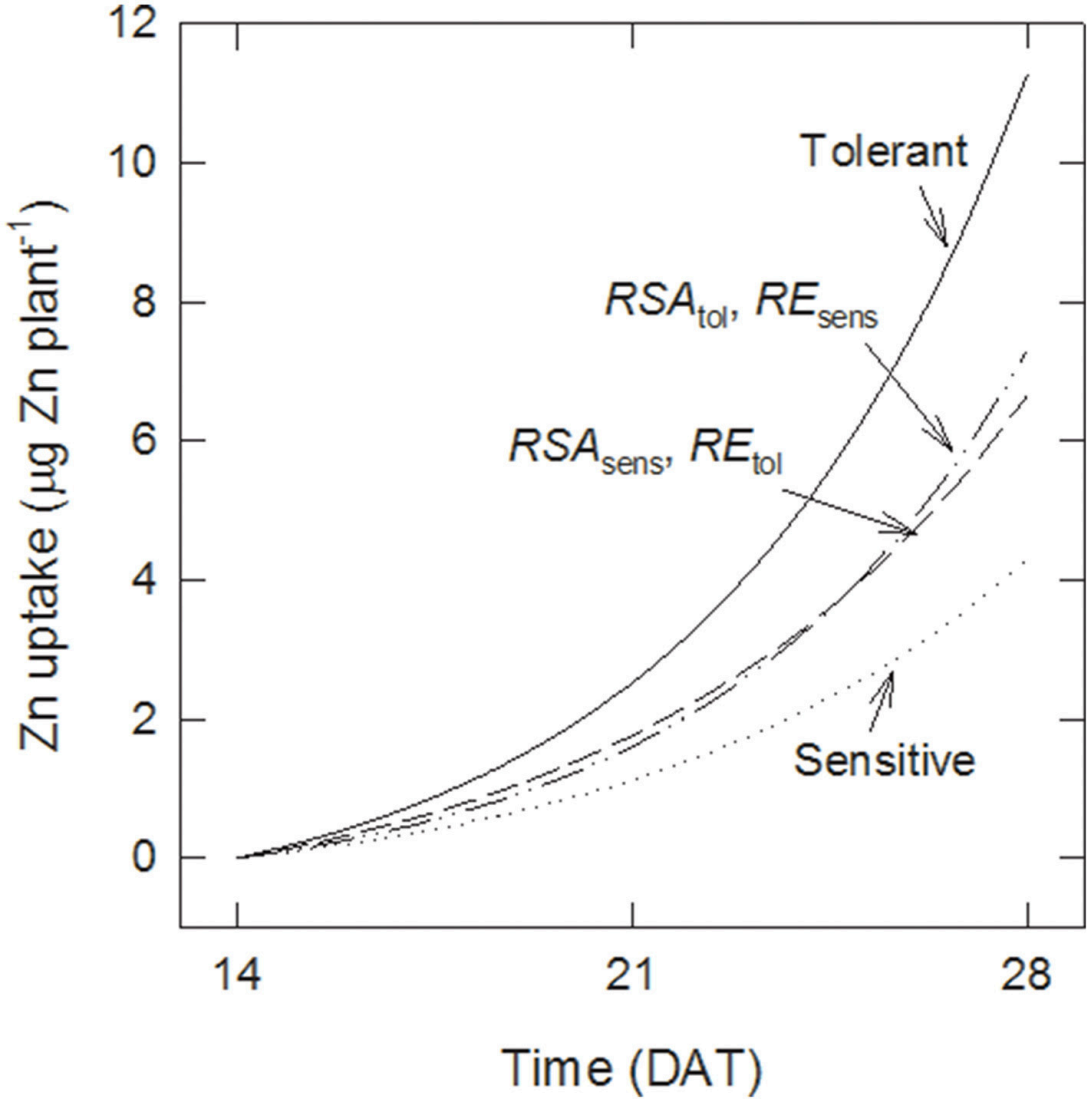
**Root surface area and uptake per unit surface area both contribute to tolerance**. Effect of increasing either root surface area (*RSA*) or root efficiency (*RE*) on Zn uptake by sensitive genotypes compared with tolerant ones calculated with Equations (5–8).

The differences in Zn uptake between tolerant and sensitive genotypes were 1.4 and 7.0 μg plant^−1^ at 21 and 28 DAT, respectively. Figure 4 shows estimates of the contribution of better root growth and root efficiency to these differences. Assuming that sensitive genotypes maintain their root development (i.e., they retain *RSA*_sens_ but take up Zn with the greater efficiency of the tolerant group, *RE*_tol_), then their uptake would increase to 1.8 and 6.7 μg plant^−1^ at 21 and 28 DAT, respectively. Thus, changes in *RE* could explain 46 to 33% of the difference between the genotype groups. Maintaining the lower *RE* but increasing root growth to the level of the tolerant genotypes (*RSA*_tol_) would have a smaller effect than *RE* at 21 DAT (35% more Zn uptake than with *RSA*_sens_) but a larger effect at 28 DAT (43% more uptake). To account for 100% of the difference between both groups a third term needs to be included, describing that the additional *RSA* of the tolerant group has the higher *RE* of the tolerant group.

### Effect of planting density

**Figure 5:**
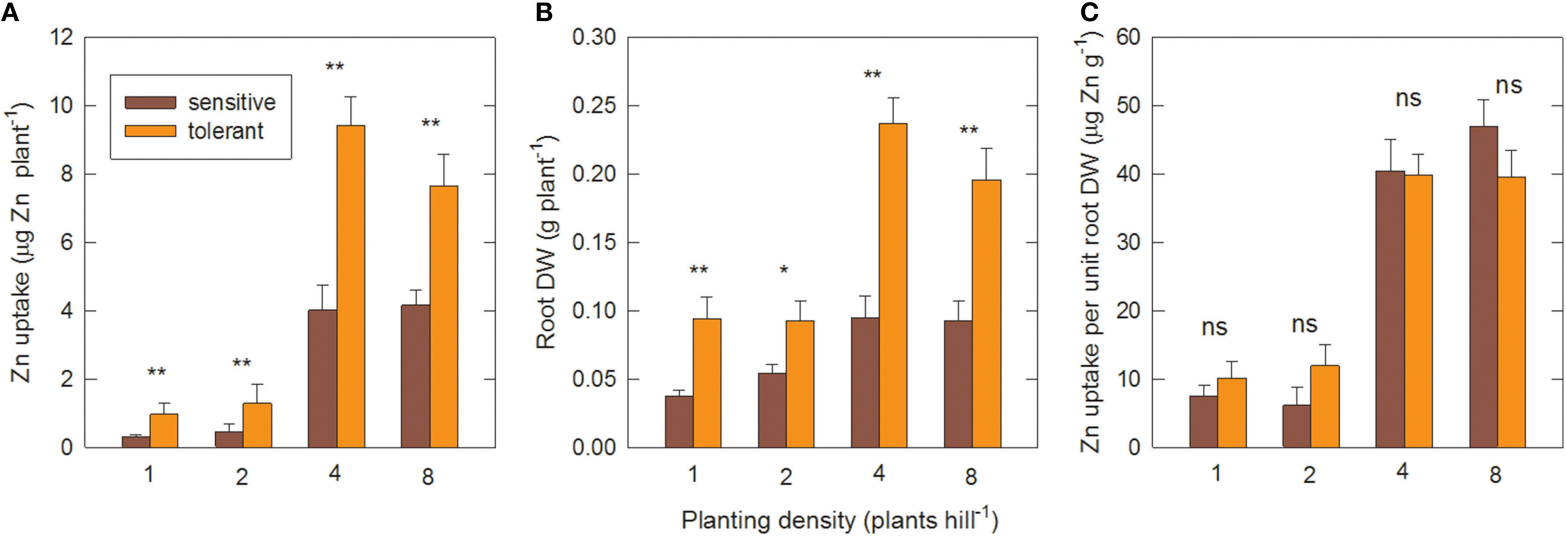
**Zinc uptake per unit root surface area increases with closer planting**. **(A)** Shoot Zn uptake per plant, **(B)** root DW per plant and **(C)** Zn uptake per unit root DW at different planting densities in tolerant and sensitive genotype groups at 28 DAT. Planting density is the number of seedlings transplanted together per hill. Uptake was calculated from shoot Zn content minus seedling Zn content. Data are means ± standard errors (*n* = 4 × number of genotypes in group). ns, ^*, **^ = genotype differences not signif., signif. at *P* ≤ 0.05, signif. at *P* ≤ 0.01.

Figure 5 shows the effect of planting density on Zn uptake and root efficiency in the genotype groups, measured in the second field experiment. When genotypes were transplanted as single seedlings per hill, or as two seedlings per hill, they remained Zn-deficient up to 28 DAT, as seen by very low Zn uptake (Figure 5A) and highly suboptimal shoot Zn concentrations of around 11 μg g^−1^ (data not shown). Transplanting four plants per hill led to about 10-fold greater Zn uptake per plant compared to single-plant hills. The proportional increase was similar in sensitive and tolerant genotypes. However, tolerant genotypes had significantly higher Zn uptake at all densities (Figure 5A). Root biomass per plant also increased in both groups when density was changed from one to four plants per hill (Figure 5B) but this 2.5-fold increase was far smaller than the increase in Zn uptake. As a result there was a 4-fold increase in root efficiency (expressed as Zn taken up per final root biomass) in four-plant hills compared to single plants (Figure 5C).

Further increasing plant density to eight plants per hill had no additional effect on Zn uptake per plant. However, the total biomass and Zn uptake per hill increased further. This increase was near-linear in the sensitive group but tolerant genotypes had reached a point of partial saturation as shown by the per plant decrease in Zn uptake and root biomass at the highest planting density (Figures 5A,B). The positive planting density effect was only observed in the –Zn treatment; in the +Zn treatment greater densities reduced biomass accumulation (Figure S3), presumably due to competition effects.

### Seedling Zn efficiency and redistribution

**Figure 6:**
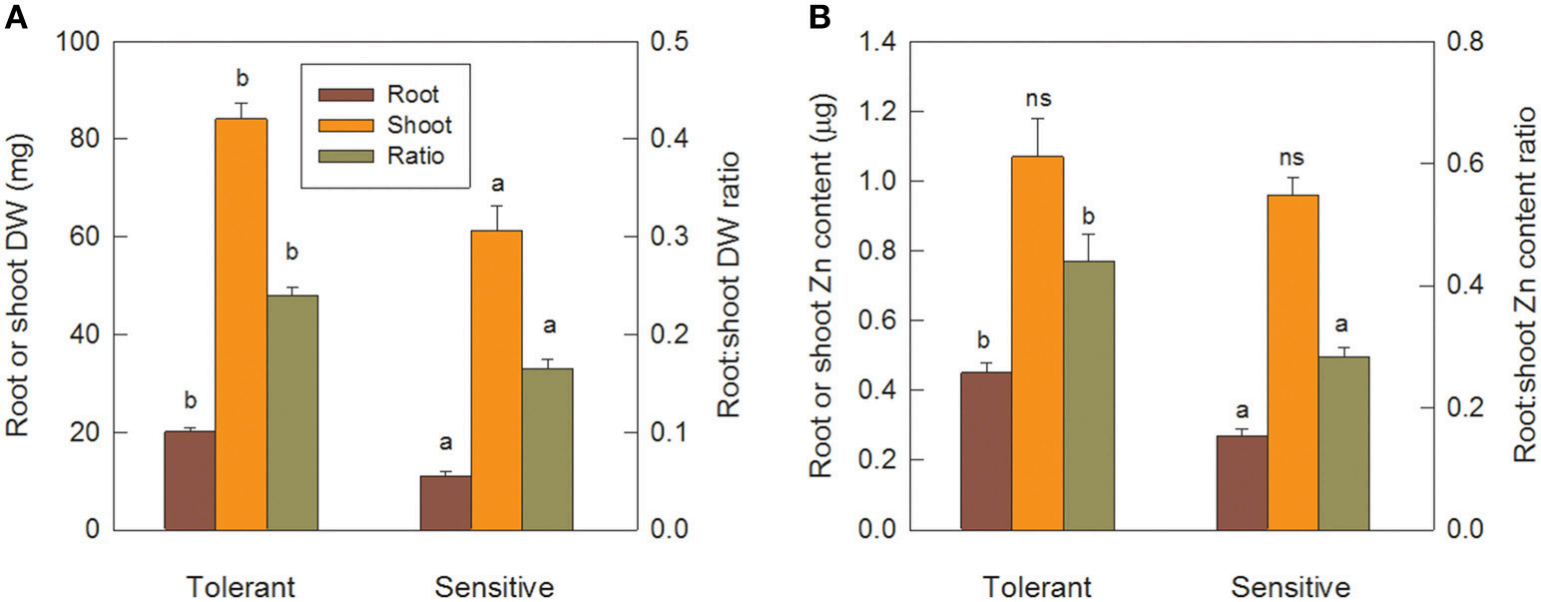
**Tolerant genotypes are better at new root initiation and remobilization of Zn from shoots to roots**. Seedlings were pre-cultured for 12 d, then roots were removed and allowed to re-grow for 13 d in Zn-free nutrient solution. Panel **(A)** shows root and shoot DW and root to shoot DW ratio. Panel **(B)** shows root and shoot Zn content and root to shoot Zn content ratio. Error bars indicate SE of individual means and letters indicate significant differences in group means (LSD *P* ≤ 0.05).

After the 12 d pre-treatment in Zn-free nutrient solution, seedlings of the sensitive and tolerant groups did not differ significantly in root or shoot biomass or respective Zn content, but total Zn content was 20% higher in the tolerant group (Table 3; data only shown for total Zn content). Re-growth of roots following their complete removal would depend on Zn re-distribution from shoots and on the efficiency of Zn utilization for root biomass production. Tolerant genotypes developed new crown roots faster than sensitive ones (Table 3) and had significantly greater root and shoot biomass (Figure 6A). Shoot Zn content did not differ between groups, but root Zn content was 66% greater in tolerant than sensitive genotypes (Figure 6B). That the advantage of tolerant over sensitive groups was more pronounced for roots than shoots was confirmed by a 45% greater root to shoot biomass ratio in the tolerant group. This increased to 55% for the Zn distribution between root and shoot (Figure 6). In contrast to Zn redistribution we did not detect significant differences in tissue Zn concentrations between the genotype groups (Table 3).

**Table 3:** **Remobilization of Zn from shoots to roots following root removal**.

	**Time (DAP)**	**Sensitive genotypes**	**Tolerant genotypes**	**LSD (*P* ≤ 0.05)**
Crown root number	0	12.4	14.0	ns
	1	2.5	3.5	0.9
	3	8.8	10.7	1.0
Total Zn content (μg)	0	1.11	1.33	0.18
	13	1.23	1.53	0.28
Root Zn concentration (μg g^−1^)	0	49.1	55.6	4.4
	13	24.7	22.6	ns
Shoot Zn concentration (μg g^−1^)	0	36.8	39.9	2.9
	13	14.4	12.5	ns

*After a 12-day pre-treatment period (time = 0 days after pre-treatment, DAP), roots were removed: one batch of seedlings was sampled for biomass and tissue Zn analysis; a second batch was then grown for an additional 13 days in Zn-free nutrient solution*.

## Discussion

### Crown root development and root efficiency are causative of Zn deficiency tolerance

We have identified two rice breeding lines tolerant to soil Zn deficiency and three sensitive to it, consistent with previous research with these genotypes on a similar soil but with less severe deficiency (Impa et al., 2013). The tolerant genotypes had greater Zn uptake from 14 DAT in the –Zn plots and a greater number of new crown roots and total root surface area per plant compared to sensitive genotypes but shoot Zn concentrations were similar. The greater increases in root number compared to Zn uptake at 14 DAT in tolerant genotypes suggests crown root development is a primary tolerance mechanism, and not just a consequence of better Zn uptake driving subsequent root growth.

Further evidence for crown root development being a primary tolerance mechanism was provided by the experiment in Zn-free nutrient solution. As Zn was not supplied, better crown root development must have been independent of new Zn uptake, and the results in Table 3 show that Zn redistribution from shoots to roots, or internal Zn utilization efficiency, or both are key tolerance mechanisms. Following the complete removal of roots, the tolerant group retranslocated 0.45 μg Zn from shoots to roots compared to only 0.27 μg Zn in the sensitive group. With this 67% greater Zn content in their roots, tolerant genotypes produced 83% greater root biomass, possibly indicating higher internal Zn utilization efficiency. However, root Zn concentrations were only marginally smaller in the tolerant group (22.6 vs. 24.7 μg Zn g^−1^), and, since this difference was not statistically significant, we conclude that Zn distribution rather than internal Zn utilization drives better root development in tolerant genotypes.

The removal of roots in this experiment simulates the root damage that seedlings suffer when pulled from the nursery and transplanted to paddy fields. While the damage in the field will be less severe, Zn uptake by surviving seedling roots will be greatly impeded and post-transplanting Zn uptake will be dominated by newly-formed crown roots. The observed delay in Zn uptake after transplanting in the field experiments (Figure 2) is consistent with this postulation. Hence better root re-growth via Zn retranslocation from shoot reserves in tolerant genotypes should contribute to tolerance under real field conditions. That we did not detect a positive association between Zn uptake and the root to shoot DW ratio in the field experiments (Table 2) does not contradict our conclusion that putting more resources (including Zn) into roots is a crucial tolerance mechanism. Tolerant genotypes did have greater root biomass. But the shift toward greater root growth may quickly become unnoticeable as bigger roots take up more Zn which supports additional shoot growth. This subsequent positive feedback effect was avoided in our nutrient culture experiment because the solution contained no Zn.

Having established that crown root development is a causal tolerance mechanism the question to be addressed next is whether higher Zn uptake was merely a subsequent effect of having higher root surface area available for uptake. This question was resolved through simulations of root efficiency (*RE*, the rate of Zn uptake per unit root surface area). The tolerant genotype group had 67% greater RE at 15 DAT and 58% at 28 DAT and in addition *RE* more than tripled over time in both groups. *RE* furthermore increased if seedlings were transplanted at densities of four or more seedlings per hill, indicating some concentration-dependent rhizosphere effect would be of importance. Thus, we conclude that tolerance of Zn deficiency is caused by two independent tolerance mechanisms. Results in Figure 4 show that better root growth and *RE* contributed roughly equally to the greater uptake by the tolerant genotypes.

Differences in *RE* might be due to differences in root absorbing power for Zn (i.e., the influx of Zn for a given Zn concentration in the soil solution at the root surface), which in turn may be caused by differences in Zn transporter activities. Two lines of evidence suggest this may not be the case. The hypothesis that genotypic differences in tolerance to Zn deficiency were caused by differential expression of key Zn transporters was tested in a transcriptomic study by Widodo et al. (2010) on root and shoot tissue sampled from the same field as used here. While many Zn transporters were up-regulated under Zn deficiency, this tended to occur equally in sensitive and tolerant genotypes, hence evidence only suggested Zn transporters were part of the stress response but not of tolerance. If greater Zn uptake was only due to high Zn transporter activity, Zn depletion zones should develop around roots over time and at higher rooting density and that would reduce *RE* over time and with increasing density. Contrary observations made here therefore suggest root-induced changes in the rhizosphere that increase Zn solubility are more likely candidate mechanisms as it has been shown that their positive effect would increase over time and with the planting density effect (Ptashnyk et al., 2011).

### Possible tolerance mechanisms

Our objective was to determine whether superior Zn uptake by tolerant rice genotypes in Zn-deficient soil is causally related to genotypic differences in crown root development or in the Zn uptake efficiency of the roots. We have shown that each type of mechanism is equally important. Tolerant genotypes produce more crown roots and greater root surface area independent of Zn uptake and the increased surface area will lead to more Zn uptake in tolerant genotypes, which in turn may allow more root growth and therefore a positive feedback loop. A second tolerance mechanism enhances Zn uptake per unit root surface area (*RE*). For breeding purposes it would be desirable to identify markers associated with both traits and to use these as quick diagnostic tools in marker aided selection. While it is beyond the scope of this study to identify such markers, we shall briefly discuss possible mechanisms underlying both causative tolerance mechanisms as this may aid in designing further experiments for marker detection.

Crown root development is a process known to be regulated by auxin and several key genes mediating this response have been identified (Inukai et al., 2005; Kitomi et al., 2008). However, none of these *Crown rootless* genes were differentially expressed between tolerant and sensitive genotypes (Widodo et al., 2010) whereas several other auxin related genes were differentially expressed between both groups. It is therefore possible that the decrease in root number observed under Zn deficiency is related to altered auxin metabolism or signaling and this will need to be investigated further. In the absence of clear candidate genes crown root development under Zn deficiency should be targeted as a trait in QTL or genome wide association studies (GWAS).

One possible rhizosphere mechanism causing higher *RE* is secretion by the roots of Zn-chelating phytosiderophores, such as deoxymugineic acid (DMA; Arnold et al., 2010; Widodo et al., 2010). DMA is thought to be an effective Zn solubilizing agent in other graminaceous species but much smaller quantities are found in secretions of rice. Ptashnyk et al. (2011) showed with a model that DMA secretion at rates indicated by Widodo et al. (2010) could provide significant extra Zn uptake to rice in a strongly Zn-deficient soil. But we have so far failed to detect significant DMA secretion from the tolerant genotypes used here (Mori, unpublished data), and we are therefore not yet able to confirm this as a key tolerance mechanism. Three other processes in the rice rhizosphere are expected to affect Zn solubility (Begg et al., 1994; Kirk and Bajita, 1995; Kirk, 2003): (1) rhizosphere oxygenation by root-released O_2_, causing oxidation of ferrous iron to ferric oxides and releasing H^+^ ions which tend to lower the pH; (2) venting of soil CO_2_ through the roots, since very large CO_2_ concentrations develop in submerged soil as respiratory CO_2_ accumulates; and (3) release of H^+^ from the roots to balance excess intake of cations (particularly ${\text{NH}}_{4}^{+}$) over anions. The venting of CO_2_, together with any decrease in rhizosphere pH, will mean the concentration of ${\text{HCO}}_{3}^{-}$ in the soil solution around roots is substantially less than in the bulk soil. A decrease in rhizosphere ${\text{HCO}}_{3}^{-}$ or pH or both will also tend to make Zn more soluble (Gao et al., 2010). Given the strongly inhibitory effects of ${\text{HCO}}_{3}^{-}$ on Zn absorption and root growth (Rose et al., 2011, 2012), we conclude CO_2_ venting is a strong candidate mechanism for Zn-deficiency tolerance. The extent of CO_2_ venting will increase with root growth and increasing plant density, consistent with our observations.

## Conclusions

Here we have used experimental field data to simulate Zn uptake in dependence of root size and these simulations have enabled us to resolve cause and effect relations with regard to tolerance of Zn deficiency. Having identified root development, possibly driven by crown root emergence, as an independent main tolerance factor, rice breeders may want to identify loci controlling this trait in order to develop linked markers to be used in their selection programs. We do not yet have sufficient understanding of rhizosphere interactions under Zn deficiency to do the same for root efficiency and a first priority would be to develop a screening protocol suited to expose phenotypic variation for the trait in order to proceed with QTL or GWAS mapping.

## Author contributions

AM, JL, MM, SJ, and MW planned and conducted field experiments and plant tissue analysis, AN planned and conducted nutrient solution experiments, AM, JL, GK, AN, and MW did the data analysis and AM, GK, SJ, and MW wrote the manuscript.

### Conflict of interest statement

The authors declare that the research was conducted in the absence of any commercial or financial relationships that could be construed as a potential conflict of interest.
