# Supplementary material for: Rice Genotype Differences in Tolerance of Zinc-Deficient Soils: Evidence for the Importance of Root-Induced Changes in the Rhizosphere
Source: Front Plant Sci. 2016 Jan 11;6:1160. doi: 10.3389/fpls.2015.01160 (PMC4707259; doi:10.3389/fpls.2015.01160)
Supplement: Supplementary file 1 [file Presentation1.pptx]

## Slide 1
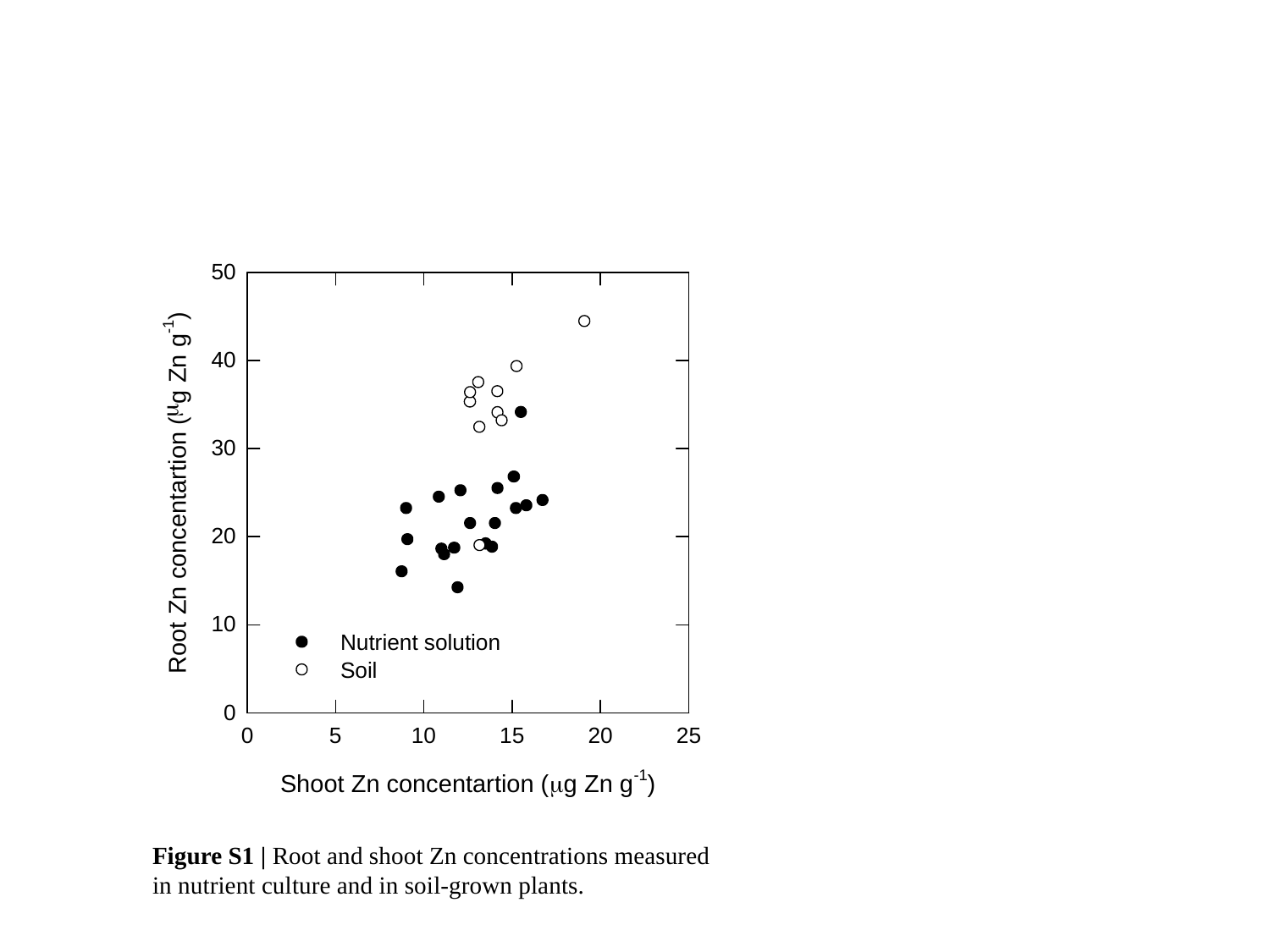

Figure S1 | Root and shoot Zn concentrations measured in nutrient culture and in soil-grown plants.
