# Supplementary material for: Rice Genotype Differences in Tolerance of Zinc-Deficient Soils: Evidence for the Importance of Root-Induced Changes in the Rhizosphere
Source: Front Plant Sci. 2016 Jan 11;6:1160. doi: 10.3389/fpls.2015.01160 (PMC4707259; doi:10.3389/fpls.2015.01160)
Supplement: Supplementary file 2 [file Presentation2.pptx]

## Slide 1
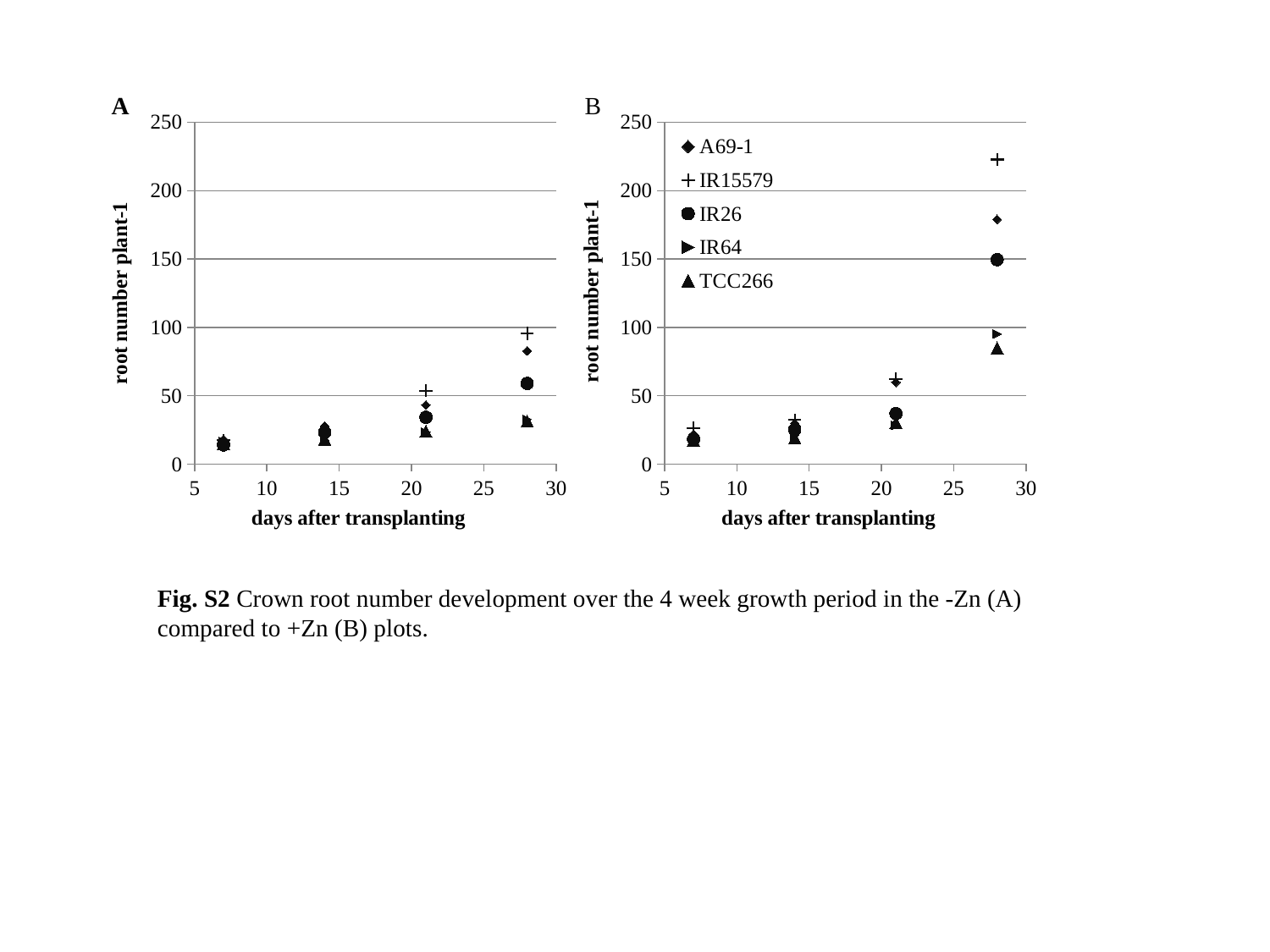

A
B
### Chart
| Category | A69-1 | IR15579 | IR26 | IR64 | TCC266 |
|---|---|---|---|---|---|
### Chart
| Category | A69-1 | IR15579 | IR26 | IR64 | TCC266 |
|---|---|---|---|---|---|Fig. S2 Crown root number development over the 4 week growth period in the -Zn (A) compared to +Zn (B) plots.
