# Supplementary material for: Rice Genotype Differences in Tolerance of Zinc-Deficient Soils: Evidence for the Importance of Root-Induced Changes in the Rhizosphere
Source: Front Plant Sci. 2016 Jan 11;6:1160. doi: 10.3389/fpls.2015.01160 (PMC4707259; doi:10.3389/fpls.2015.01160)
Supplement: Supplementary file 3 [file Presentation3.pptx]

## Slide 1
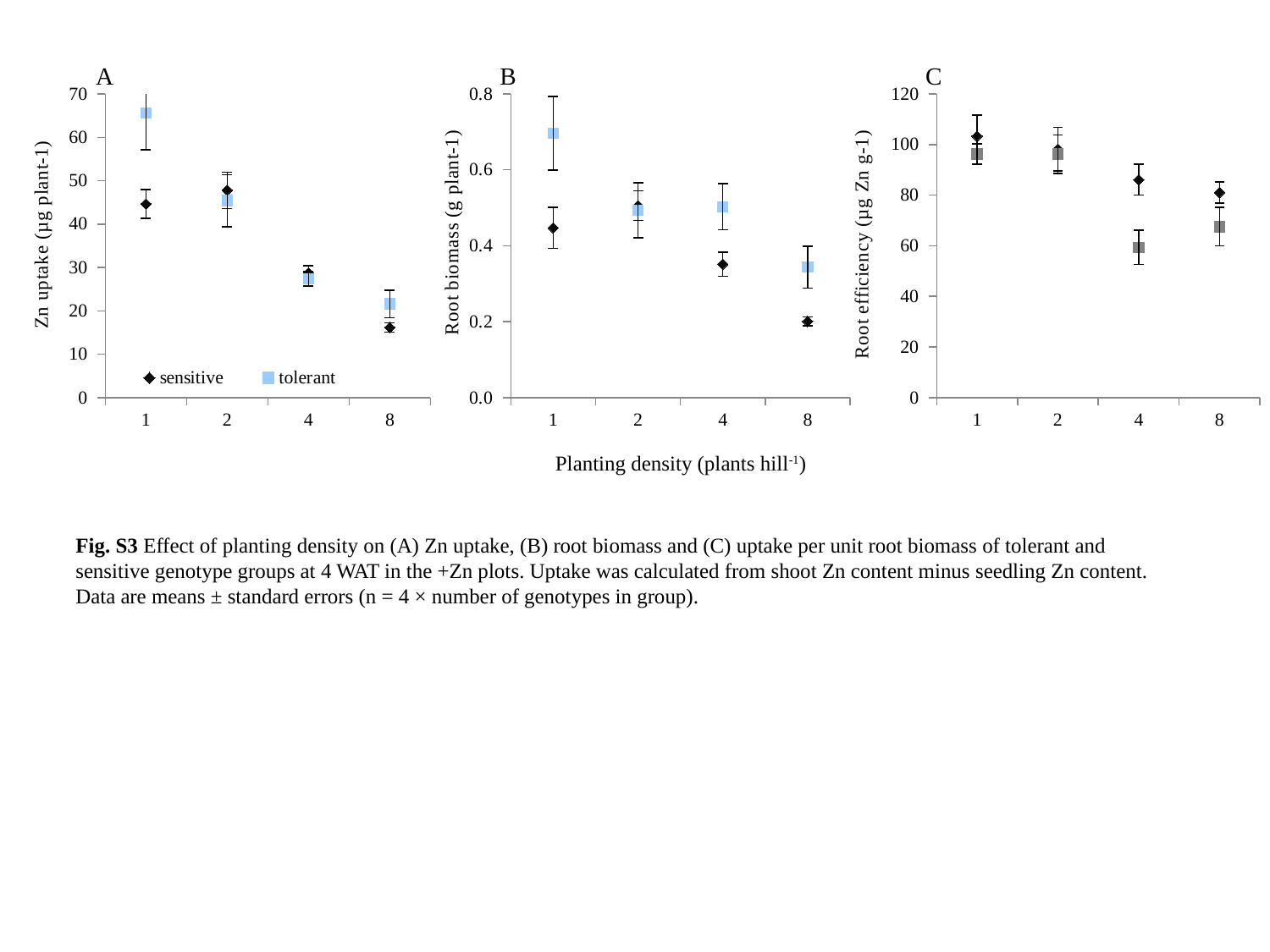

A
B
C
### Chart
| Category | sensitive | tolerant |
|---|---|---|
| 1 | 44.652 | 65.629 |
| 2 | 47.803 | 45.386 |
| 4 | 28.724 | 27.413 |
| 8 | 16.163 | 21.604 |
### Chart
| Category | Sensitive | Tolerant |
|---|---|---|
| 1 | 0.4475 | 0.6963 |
| 2 | 0.5058 | 0.4938 |
| 4 | 0.3517 | 0.5025 |
| 8 | 0.2008 | 0.3438 |
### Chart
| Category | Sensitive | Tolerant |
|---|---|---|
| 1 | 103.28 | 96.229 |
| 2 | 98.221 | 96.21 |
| 4 | 86.126 | 59.382 |
| 8 | 81.047 | 67.594 |Planting density (plants hill-1)
Fig. S3 Effect of planting density on (A) Zn uptake, (B) root biomass and (C) uptake per unit root biomass of tolerant and sensitive genotype groups at 4 WAT in the +Zn plots. Uptake was calculated from shoot Zn content minus seedling Zn content. Data are means ± standard errors (n = 4 × number of genotypes in group).
